# Mapping multilevel barriers impacting the performance of Ethiopia’s Community-Based Health Extension Program: A scoping review

**DOI:** 10.1371/journal.pone.0351989

**Published:** 2026-07-20

**Authors:** Kora Tushune Godana, Zewdie Birhanu, Gugsa Nemera, Kasahun Eba, Fikadu Balcha, Yibeltal Assefa, Wim V. Damme, Peter Decat

**Affiliations:** 1 Department of Health Policy and Management, Public Health Faculty, Jimma University Jimma, Jimma, Ethiopia; 2 Department of Health, Behavior and Society, Faculty of Public Health, Jimma University, Jimma, Ethiopia; 3 Department of Nursing, Faculty of Health Sciences, Jimma University, Jimma, Ethiopia; 4 Department of Environmental Health Sciences and Technology, Faculty of Public Health, Jimma University, Jimma, Ethiopia; 5 School of Public Health, The University of Queensland, Brisbane, Australia; 6 Department of Public Health, Institute of Tropical Medicine, Antwerp, Belgium; 7 Department of Public Health and Primary Care, Faculty of medicine and Health Sciences, University of Gent, Ghent, Belgium; Wollega University, ETHIOPIA

## Abstract

**Background:**

Ethiopia’s Health Extension Program (HEP) aims to improve primary care access through community-based delivery of essential preventive and promotive services. Despite progress, some health outcomes remain suboptimal, underscoring the need to optimize HEP implementation. Identifying barriers of the HEP and its Health Extension Workers (HEWs) is critical for enhancing program effectiveness. This scoping review aims to identify and synthesize evidence on the barriers impacting the performance of the Ethiopian HEP and HEWs.

**Methods:**

A comprehensive search strategy was conducted across multiple databases, including PubMed, EMBASE, Web of Science, and relevant institutional websites, as well as hand searches via Google Scholar and Google engine, to identify both published and unpublished documents (documents (government reports and policy documents (strategic plans, program reviews, annual performance reports), development partner publications, doctoral dissertations, and masters theses) related to the HEP. The review encompassed studies published since January 2003. Data extraction was performed using Atlas.ti software version 7.5.18, which facilitated the collection of verbatim excerpts and the application of thematic coding to highlight key findings. The results are organized and presented through thematic analysis, focusing on multi-dimensional barriers and challenges.

**Results:**

The review reveals multi-dimensional barriers, including a misalignment between the HEP’s preventive design and the community’s demand for curative services, which undermines community trust and program acceptance, and gender-imbalanced workforce, which creates barriers to service delivery in certain cultural contexts. Additional barriers include a lack of training and retention strategies, and digital literacy and access gaps which further impede effectiveness of the program. Excessive workloads, non-health tasks, and poor living conditions, divert HEW’s focus from their primary responsibilities. Weak community engagement, inadequate integration of Traditional Birth Attendants (TBAs), and inefficiencies in model family development limit the program’s impact. Misconceptions in the community, resource constraints, fragmented stakeholder interactions, ineffective supervision, governance issues, and political interference directly impede performance.

**Conclusion:**

The HEP faces deeply entrenched, multi-dimensional barriers that limit its potential to achieve optimal health outcomes. Key policy recommendations include integrating curative services, ensuring a balanced gender mix in the workforce, improving working conditions and expanding community engagement. Further research is needed to quantify barriers, assess their impact, and identify key influencing factors, with stakeholder engagement and implementation research being essential for developing effective solutions.

## Introduction

The Health Extension Program (HEP) is a community-based primary healthcare initiative established by the Ethiopian government in 2003 to enhance healthcare accessibility and outcomes in rural and remote regions [1]. Focusing on essential preventive and promotive care [2,3], the HEP has played a pivotal role in achieving Ethiopia’s health-related Millennium Development Goals [4].

Despite widespread recognition of the HEP as a critical component for advancing health-related indicators, empirical evidence reveals systemic and operational barriers impeding its performance. For instance, one study reported barriers related to Health Extension Worker (HEWs) productivity working environment, and living conditions in addition to capacity of health posts and, social determinants of health [5]. Several other research works identified factors like insufficient medical equipment and pharmaceutical supplies, limited supervision, the absence of a robust referral system, high turnover rates among HEWs, a lack of clear career progression for HEWs, uncompetitive salary structures, and the constrained scope of the Health Extension Program (HEP) as barriers to its efficacy [6–8]. Furthermore, studies have highlighted that HEWs often lack essential skills due to limited support and that socioeconomic barriers impede access to healthcare services, particularly for marginalized groups [9,10].

Recognizing these challenges, the Ethiopian government, in collaboration with development partners, has implemented various initiatives to strengthen the HEP and support its HEWs. These efforts have included periodic revisions of the HEP training curriculum, the introduction of performance-based incentives, and the provision of motorcycle ambulances to improve access in hard-to-reach areas [8,11]. The Health Sector Transformation Plans (HSTP I and II) have also prioritized HEP revitalization through initiatives such as the “HEP 2.0” strategy, which aimed to enhance the technical capacity of HEWs, strengthen the referral system, and improve the supply chain at the health post level [12,13]. Additionally, the government has expanded the Urban Health Extension Program to address the unique needs of urban populations and has piloted digital health information systems to improve data quality and reporting [14]. Despite these concerted efforts, persistent and emerging barriers continue to undermine the HEP performance. However, to the authors’ knowledge there is no study that investigates a multi-level analysis of the interconnected barriers that impede HEP performance.

The program’s heavy reliance on community-based workers can create barriers to consistent service delivery and inconsistent quality of care, as evident in previous research [15,16]. Furthermore, the HEP emphasis on preventive and promotive health services may inadvertently create barriers to curative services, ultimately compromising the provision of adequate care to individuals with pressing health needs, which may exacerbate existing health disparities and undermine the overall effectiveness of the program [17].

Given these persisting gaps, effectively mapping the multi-level barriers is pivotal to ensuring the long-term viability and impact of the HEP. This could help in mitigating the obstacles for optimizing the program’s operational efficiency, efficacy, and equity, thereby making a substantive contribution to the realization of universal health coverage and the attainment of the Sustainable Development Goals (SDGs) in Ethiopia. While previous research has examined the performance of HEP in Ethiopia, a significant knowledge gap remains in the systematic identification and mapping of the multilevel barriers that impede the optimal functioning of Ethiopian HEP, highlighting the necessity for a comprehensive examination of these interconnected factors. Understanding these barriers is crucial for identifying targeted interventions to address them and optimize the program’s performance.

A compelling rationale for this scoping review is the fragmented nature of evidence on barriers to HEP. While numerous studies identify challenges affecting the program, they typically focus on isolated issues such as health worker motivation, supply chain gaps, or community factors within specific geographic areas. This approach fails to capture the complex, interconnected obstacles that Health Extension Workers (HEWs) and the program face. For example, high HEW turnover likely emerges from interrelated factors including inadequate training, poor living conditions, limited career progression, and community dissatisfaction, yet no study has synthesized these multi-level interactions comprehensively.

A scoping review is the most appropriate methodology to address this gap, as it is designed to map the breadth of literature on broad topics and synthesize diverse evidence types including qualitative studies, quantitative surveys, and grey literature. This approach will enable systematic identification and categorization of barriers across individual, community, health system, and policy levels while revealing their interconnectedness. By mapping existing evidence and identifying knowledge gaps, this review will provide policymakers with a cohesive understanding needed to design holistic interventions and offer researchers a clear roadmap for future implementation studies aimed at optimizing HEP performance.

This scoping review seeks to fill this gap by systematically mapping and synthesizing the available evidence to provide a holistic understanding of the barriers affecting the HEP and HEWs, thereby informing targeted and effective policy interventions.

### Review question

What are the barriers identified in local literature that hinder the overall performance of the HEP in Ethiopia.

## Methods

This study forms part of a comprehensive scoping review that examined factors influencing the Ethiopian HEP performance. The current analysis specifically examines the barriers hindering the success of HEP. We employed a structured approach based on the Joanna Briggs Institute (JBI) methodology for scoping reviews [11]. JBI utilizes the Population, Concept, and Context (PCC) framework to systematically identify and map available evidence [18] related to the target populations involved in or impacted by the HEP, key concepts related to the performance of HEP and the contexts or settings relevant to this review.

### Inclusion and exclusion criteria

This review included studies published in English from January 2003 to January 2024 and involved HEWs, community members, community groups, healthcare professionals, health managers, policymakers, and program partners to identify factors that hinders the HEP performances. In addition, government reports, policy documents, dissertations and thesis were included. Studies published in non-English language and those focused exclusively on other healthcare providers other than health extension workers (HEWs) and health administrative personnel were excluded.

### Concept

The primary concept interest in this scoping review was barriers of HEP performance in Ethiopia. Specifically, the focus was on identifying barriers and challenges related to infrastructure, facilities, financial resources, leadership and governance, support systems, work environment, and the living conditions of HEWs.

### Context

This scoping review examined documents related to HEP, including the Urban Health Extension Program, from all administrative regions and city administrations within Ethiopia.

### Types of sources

This scoping review synthesized evidence from methodologically diverse body of literature, encompassing peer-reviewed articles included experimental studies (randomized and non-randomized controlled trials, before-and-after studies, and interrupted time series analyses), observational designs (case series, individual case reports, and descriptive cross-sectional studies). Qualitative evidence was drawn from studies employing phenomenology, grounded theory, ethnography, qualitative description, action research, and feminist research frameworks. Grey literature including reports, discussion papers, and systematic reviews pertaining to the HEP and HEWs, were also included, with a particular emphasis on identifying barriers to implementation and performance.

### Search strategy

A three-step approach was employed to identify both published and unpublished documents pertaining to the Ethiopian HEPs from January 2003 to January 2024. The search was restricted to studies published from January 2003 onwards, coinciding with the official launch of the Health Extension Program, to ensure all captured literature pertains to the period of the program’s existence. First, an initial, limited search was conducted in MEDLINE (PubMed) and EMBASE to identify relevant articles. The text words along with the index terms used to describe the titles and abstracts of these articles were analyzed to develop a comprehensive search strategy for PubMed (see Appendix I). This search strategy, including all identified keywords and index terms, was then tailored for each database and information source included in the review. Additionally, the reference lists of all included sources of evidence were screened to identify further relevant studies. The databases searched were PubMed, EMBASE, Web of Science, Cochrane Database of Systematic Reviews, JBI Evidence Synthesis, and African Journals Online (AJOL). To identify unpublished studies and grey literature, searches were conducted in ProQuest Dissertations and Theses, Google Scholar, and institutional websites such as those of Jimma University, Addis Ababa University, the Ethiopian Public Health Institute, and the Ministry of Health.

### Study/source of evidence selection

After completing the search, all identified citations were compiled and uploaded into EndNote version 20 (Clarivate Analytics, PA, USA), where duplicates were removed. Following a pilot test, the titles and abstracts were screened by five independent reviewers to assess their relevance against the inclusion criteria for the review. Sources deemed potentially relevant were retrieved in full, and their citation details were imported into Atlas ti version 7.5.18 (GmbH, Berlin, Germany) for the extraction of study characteristics and key findings. The full text of selected citations was thoroughly evaluated against the inclusion criteria by three or more independent reviewers. Reasons for excluding sources that did not meet the inclusion criteria at the full-text stage were documented and reported in the scoping review. Any disagreements that arose between reviewers during each stage of the selection process were resolved through discussion or with the involvement of an additional reviewer. The results of the search and the study inclusion process were fully detailed in the final scoping review and presented in a Preferred Reporting Items for Systematic Reviews and Meta-Analyses extension for scoping reviews (PRISMA-ScR) flow diagram [19] (see S3 File: Preferred Reporting Items for Systematic reviews and Meta-Analyses extension for Scoping Reviews (PRISMA-ScR) Checklist

### Data analysis and presentation

The results of the scoping review are organized and presented using thematic and sub-theme analysis, supported by narrative descriptions of the findings extracted from the included papers. The thematic areas are aligned with the objectives of the scoping review, highlighting and characterizing the multi-dimensional barriers that negatively affected the performance of the HEP. Finally, these multi-dimensional themes are conceptualized and mapped in a diagram that illustrates a pathway to enhanced performance.

## Results

### Data extraction

Data were extracted by four independent reviewers (KT, KE, GN, FB) following the JBI guidance for conducting scoping reviews using standardized data extraction form of excel sheet adapted from JBI. The extraction tool captures specific details about study characteristics, methods, participants, concepts, context, and key findings relevant to the review questions (Supplementary material Data extraction template). The extraction tool was tested on five studies and was refined accordingly. The disagreements between the reviewers were addressed by discussion among the reviewers based on consensus with additional insights from ZB & SA. The extraction process was supported by Atlas.ti software version 7.5.18, which was used to capture segments of the papers as quotations, providing verbatim excerpts that illustrate themes, concepts, or findings within the data. Additionally, we used codes; words or short phrases that symbolically assign essence-capturing meaning; to reference the selected quotations.

The draft data extraction tool was modified and refined as needed throughout the process of extracting data from each included source of evidence. Any disagreements between reviewers were resolved through discussion or with the involvement of an additional reviewer. When necessary, the authors of papers were contacted to request missing or additional data.

### Study selection

**Fig 1:**
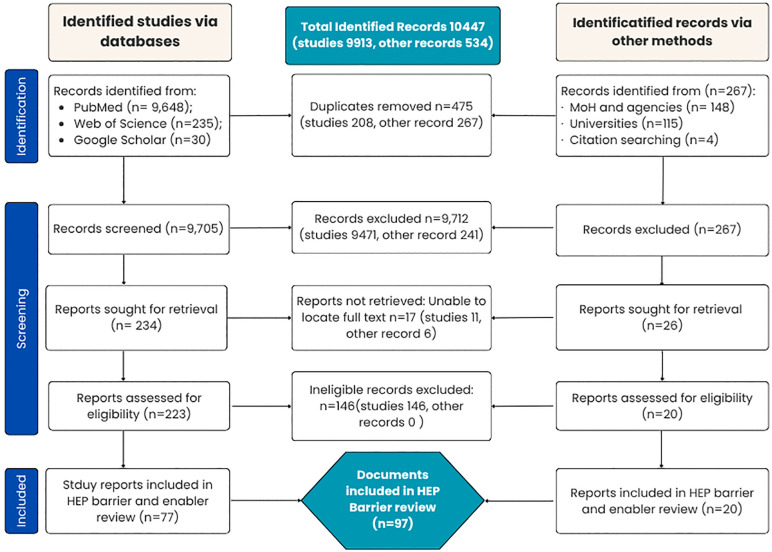
PRSIMA flow chart.

A total of 10,447 records (9,913 studies from databases and 534 from other sources) were identified. After the removal of 475 duplicates; 9,705 studies and 267 other records were screened; and 9,875 were excluded based on abstract; full-text and eligibility screening resulting in 97 records included in the review for analysis (S1 File: search strategy). The search results and study selection process are summarized in **Fig 1**.

### Characteristics of included documents

The review encompassed 97 documents addressing the barriers and challenges affecting the performance of HEWs/HEP in Ethiopia. Among these documents, the majority were published articles (n = 72), followed by grey literature (n = 15) and theses (n = 10). Various study designs were represented in the included documents. Observational studies were the most common, with 44 studies, followed by 14 qualitative studies, 9 experimental/interventional studies, 16 policy document analyses, 10 reviews and 4 studies with a mixed-methods approach.

The studies spanned different settings, with a community-based focus being the most frequent (33 studies) followed by facility-based settings (24 studies), studies with system-level (n = 7), and mixed settings (n = 17) were also represented, 16 studies not specific to a particular urban or rural setting. In terms of study context, rural settings were the primary focus, appearing in 47 studies, while mixed urban and rural settings were explored in 21 studies. Urban-only contexts were relatively rare, appearing in only 7 studies. An additional 18 studies were not specific to a particular urban or rural context and 4 studies did not specify the setting context.

Lastly, the population focus of the studies varied widely. Forty-three studies used mixed subjects, including HEWs, healthcare professionals, households, child care takers and community members. Twenty-seven studies focused specifically on HEWs and healthcare professionals while, 22 studies used different types of documents as data sources and 5 studies did not specify the population.

### Barriers to HEP Performance

Based on the thematic analysis of the included studies, four major thematic areas were established that encompass the major barriers to HEP performance. The themes, as discussed in detail below, include: program design limitations in service scope; HEWs capacity, motivation and retention crisis; eroded community engagement and support structure; and systemic failures in coordination, supervision, and support (see S2 File: Barrier Grand Summary Table).

#### 1. Program design limitations in service scope.

A major barrier was the misalignment between HEPs’ focus on prevention and community’s’ demand for curative services. This disconnects eroded trust and acceptance of health services [12,20–24]. The discrepancy was particularly pronounced in urban settings, where residents expected urban hea;th extension workers (UHEWs) to provide clinical care for conditions such as eye diseases, tuberculosis, diarrheal illnesses, and internal organ problems, rather than exclusively delivering health education [23–25]. This expectation gap was reflected in quantitative findings; nearly half (48.8%) of respondents in one study expressed dissatisfaction with HEW services, which subsequently contributed to the avoidance of health posts visits [24,25]. Furthermore, limited community awareness regarding the goals and objectives the HEP further exacerbated the erosion of community trust [12,24,26,27].

The female-only composition of the HEW workforce’s created multiple barriers. First, maternity leave resulted in disruptions to service continuity at health posts [28,29]. Second, safety concerns limited female HEWs’ capacity to provide nighttime care for laboring mothers in remote or pastoralist areas [7,25,30]. Third, while female HEWs were preferred by women for confidential health matters, the absence of male HEWs posed challenges for physically demanding tasks such as transporting commodities and logistics [15,25,31]. Furthermore, the program’s predominant focus on women as primary beneficiaries meant that men and youth were inadequately engaged. This limited engagement contributes to community dissatisfaction, low attendance at meetings, resistance to the program, and an increased burden on HEWs [12,20,22,24,32,33].

#### 2. HEWs capacity, motivation and retention crisis.

The performance of the HEP was substantially constrained by systemic challenges related to HEW capacity, motivation, and retention.

***Training and competency gaps:*** Although the duration of HEP training evolved over time, inadequate training and persistent competency gaps were consistently reported across multiple studies. The initial basic pre-service training of 12 months for rural HEWs and 3 months for UHEWs was often described as insufficient, particularly lacking adequate practical exposure [6,34,35]. For instance, one study reported 67.9% of HEWs felt their duties required more training than they had received [20].

HEW deployment orientation and job descriptions were inconsistent across regions [10,27]. Some regions provided a three-day orientation accompanied by a job description prior to deployment [2] whereas other regions, such as Oromia, Amhara, and SNNP, did not provide such orientation. Furthermore, in-service training programs were poorly coordinated and ineffective in enhancing HEW capacities and skills [20,24,36,37].

Competency gaps were evident across multiple domains, including community problem diagnosis, communication, planning, data management, and time management [38–41]*.* In some instances, these gaps led to data falsification. One study reported certain HEWs resorted to exaggerating the number of births or reclassifying neonatal deaths as stillbirths under pressure to produce favorable reports [39].

Digital literacy among HEWs was low. For instance, one study reported that only 41.5% of HEWs possessed adequate digital health literacy [42]. Multiple factors hindered digital health adoption within the HEP, including non-user-friendly digital formats, English language barrier, concerns about phone loss, power failures in rural areas, and poor connectivity [38,42].

***Workload and role creep:*** Overwhelming workload and role creep were pervasive issues. HEWs were responsible for 18 HEP packages, household visits, vertical campaigns, outreach services, and multiple registers. These responsibilities were compounded by participation in non‑health activities such as political tasks, agricultural extension duties, and community meetings [3,5,6,14,20,21,25,30,35,37,43–50]. One study found that 59.3% of HEWs rated their workload as excessive [20].

The addition of Community‑Based Health Insurance (CBHI) tasks, such as enrollment, premium collection, and identification card distribution, represents an extra burden not formally part of HEP packages, and these tasks disrupted routine services [37,51]. Additionally, campaigns often redirected HEWs away from planned activities [6].

***Living and working conditions:*** Poor living and working conditions further strained HEWs performance. Lack of safe water, electricity, adequate housing, and workplace security forced many HEWs to reside at a distance from health posts. This distance incurred transportation costs and reduced availability during standard hours, weekends, and night shifts [12,24,27,43,44,49,52–54]. This irregular presence, compounded by family interests and remote family residences, eroded community trust [12,49,52,53,55].

***Motivation and retention failures:*** Motivation and retention failures were driven by multiple factors, including low salaries, a lack of financial and non‑financial incentives, the absence of career progression pathways, and limited recognition [12,20,23,24,28,30,35,36,38,52,53,56–58]. High turnover rates were associated with heavy workload, [7,20,59] low salary assignment to non-HEP tasks, maternity leave [7,25,28], lack of transfer opportunities another healthcare facilities, denial of annual leave, living separately with families, conflicts with officials, poor supervision, competing interest with child care and better employment opportunities [7,12,25,28,30,52,53,58,60,61]. One study noted that 43.2% of HEWs felt their efforts were unproductive by organizational shortcomings in incentives and capacity development [20].

#### 3. Eroded community engagement.

The decline or deterioration of community and support structures, including village administrations, women’s groups, and the Women Development Army (WDA) and the Health Development Army (HDA), in recent years weakened program reach [32,36].

***Systemic inefficiencies in the HDA and WDA:*** Systemic inefficiencies within the HDA and WDA structures were identified, including unstandardized selection processes, a lack of role-model behavior, and a failure to influence peer networks [12,24,27,54]. WDA members faced overburdening, inadequate training, lack of supervision, and high attrition, which undermined their effectiveness as peer supporters [12,24,27,36,54,62]. Furthermore, model family training suffered from inconsistent delivery, insufficient support, and limited capacity to influence neighboring households, thereby reducing its intended impact [12,20,54,56,63,64].

***Weak integration of traditional birth attendants:*** Weak integration of Traditional Birth Attendants (TBAs) created a disconnect between formal and traditional care systems. TBAs remained trusted providers, especially for maternal and child health, while collaboration between TBAs and HEWs was poor. This lack of integration led to missed opportunities for service coordination and resulted in suboptimal service utilization, as the community’s continued reliance on TBAs negatively impacted HEP performance [21,65,66].

***Community attitudes:*** Community attitudes and resource constraints further impeded success. Mistrust of HEWs, misconceptions about the HEP program, and dissatisfaction with limited curative services contributed to community resistance and low attendance at health activities [12,20,22,24,27,32,36,43,54,65]. Inappropriate strategies, such as coercive tactics to promote institutional delivery, further damaged relationships between HEWs and the community [33,65,67].

Additionally, households often lacked the resources to act on health advice; for example, affording materials for latrine construction was a major barrier [24,68]. Deep‑seated cultural beliefs and the influence of elders (e.g., grandmothers or mothers-in-law) also hindered the adoption of promoted behaviors [21,24,47].

#### 4. Systemic failures in coordination, supervision, and support.

***Fragmented partner engagement:*** Fragmented partner engagement substantially overwhelmed HEWs. Multiple NGOs, development partners, and government programs engaged HEWs independently without coordination [20,21,24], resulting in conflicting instructions, redundant reporting requirements, and excessive task demands [47,56,66,69]. The absence of a unified engagement platform at the district, primary health care unit (PHCU), or community level [20,21,24] exacerbated confusion and stress among HEWs [66].

***Weak coordination and referral systems:*** Weak coordination and referral processes within the PHCU were evident. Information exchange and communication between HEWs, health center staff, and district management were suboptimal, and referral processes lacked clarity [5,7,27]. Competing demands originating from different levels of the health system further hindered HEWs’ ability to prioritize their duties [5,6].

***Ineffective supervision and monitoring:*** Ineffective supervision and monitoring were chronic issues. Supervisions were irregular and inconsistent and often lacked constructive feedback. Moreover, there were abusive behaviors from supervisors were also reported [5,7,23,24,29,30,37,43,44,59,64,70–73]. The performance appraisal system was perceived by HEWs as complex and fault‑finding, and it lacked community involvement or transparency and accountability. These characteristics demotivated HEWs and undermined their performance [5,24,74,75].

***Resource and infrastructure deficits:*** Resource and infrastructure deficits severely constrained HEP service delivery. Several studies reported that persistent stock‑outs of essential drugs, vaccines, contraceptives, and supplies at health posts were common [40,43,52,53,76]. Basic office furniture, stationery, storage, communication tools, and educational materials (guidelines, job aids) were often absent [12,24,30,40,41,77], which affected HEWs’ morale and job satisfaction [5]. Resource scarcity at the health post level creates feelings of frustration, helplessness, and a sense of being unsupported among HEWs and fundamentally undermines the HEPs’ sustainability [5,78].

Additionally, health posts lacked operational budgets for transportation and other costs, and inadequate roads and ambulance services hindered access to hard‑to‑reach areas [6,7,20,27,30,47,52,77,79–89].

***Governance and leadership gaps:*** Governance and leadership gaps were evident throughout the program. Government apathy characterized by a lack of sustained political commitment and insufficient funding from local to national levels undermined progress [5,12,27,30,35,37,43,47,49,53,66,71,90–92]. Leadership at district and facility levels often lacked the skills to effectively support HEWs, and political interference (e.g., involvement in elections) diverted HEWs from their health roles [24,30,55,66].

**Fig 2:**
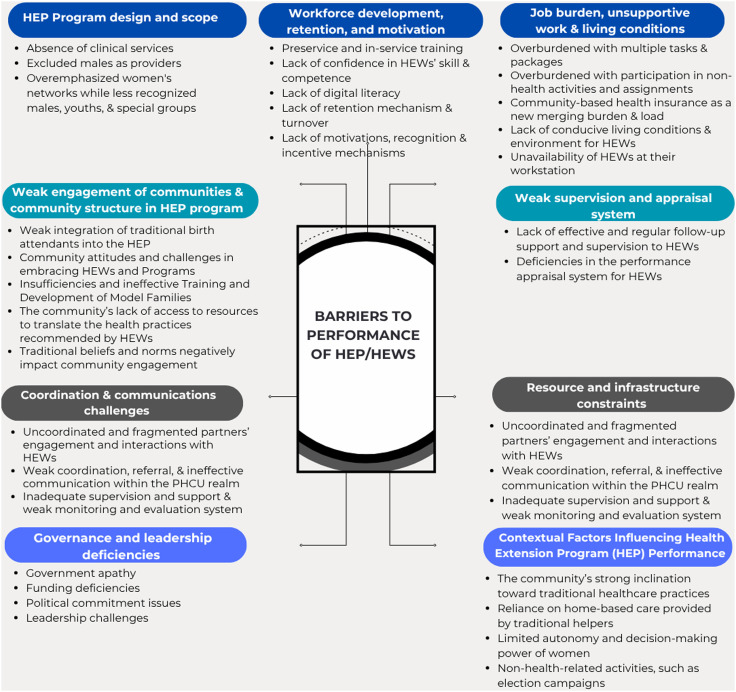
Barriers to performance of HEP.

***Contextual factors:*** Contextual factors further shaped HEW performance. These include reliance on traditional healthcare, limited women’s autonomy, socio‑economic barriers (e.g., transport costs), and challenging geographical conditions [20,21,24,33,65,66,81]. These factors were interwoven with the systemic barriers described above. Collectively, these barriers impede the performance of HEWs and the HEP, as highlighted in Fig 2.

## Discussion

This scoping review mapped the multidimensional barriers to the performance of Ethiopia’s Health Extension Program (HEP) and its Health Extension Workers (HEWs). The findings were synthesized into four interconnected thematic areas: Program design limitations in service scope and target populations; HEWs’ capacity, motivation, and retention challenges; eroded community engagement and support structure; and systematic failures in coordination, supervision, and support.

A key barrier identified was the misalignment between the HEP’s preventive design and the community’s demand for curative services. This mismatch was particularly evident in studies conducted in urban contexts, where residents expected clinical care rather than health education alone. Such disconnection undermined community trust, led to community resistance, and reduced HEP service utilization [12,20–24]. Similar tensions have been documented in community health worker (CHW) programs in India, where Accredited Social Health Activists (ASHAs) faced credibility challenges when they could not provide basic curative services [93,94]. In rural Uganda and Kenya, CHWs who offered integrated community case management (iCCM) for common childhood illnesses gained higher community acceptance than those limited to prevention [95,96]. This suggests that incorporating basic curative services or establishing clear, functional referral pathways may be essential for maintaining community trust.

The female‑only composition of the HEW workforce also emerged as a design‑level issue. While the all‑female cadre was intended to enhance the acceptability of maternal and child health services, it created unintended barriers, including service disruptions during maternity leave, safety concerns in remote areas, and difficulties with physically demanding tasks [7,25,28–30]. Moreover, the program’s heavy focus on women as beneficiaries excluded men and youth, contributing to low engagement and dissatisfaction [12,31,32,47]. Evidence from other CHW programs underscores the value of gender‑balanced teams, in which male CHWs can facilitate outreach to male community members, share physical workloads, and improve overall coverage [97–100]. A more gender‑diverse workforce may also challenge traditional norms and expand program reach [101]. Hence, to strengthen the existing HEP workforces, it is essential to recruit male HEWs to complement the female cadre, thereby enhancing operational safety, service continuity, and the reach of community engagement.

The review exposed a workforce challenge in which a systemic cycle of inadequate training, excessive workload, and poor living conditions fuels low motivation and retention, ultimately compromising HEP performance. Inadequate pre‑service and in‑service training left HEWs ill‑prepared for their roles, with gaps in clinical, data management, and digital skills [6,34,35,38–42]. Similar deficits are reported in CHW programs across LMICs, where insufficient training leads to poor-quality care and low confidence [102–104]. The added burden of non‑health tasks (political campaigns, agricultural activities, CBHI administration) and the sheer volume of HEP 18 packages and reporting requirements created unsustainable workloads [5,6,20,35]. This mirrors the findings from Uganda, Bangladesh, and South Africa, where role overload is a primary driver of burnout and turnover [105–107]. Time‑motion studies in Ethiopia and elsewhere confirm that CHWs spend substantial time on non‑core activities, reducing their availability for essential health services [46,108]. Poor living conditions at health posts (lack of water, electricity, housing, and security) forced many HEWs to live away from their workstations, leading to irregular availability and eroded community trust [12,24,27,43,49,52–54]. Similar challenges are documented in rural CHW programmes in Mozambique and Tanzania [109,110]. Motivation and retention suffered from low salaries, absence of financial and non‑financial incentives, and limited career progression, further fueled turnover [12,28,52,61]. The cumulative effect is high attrition, which disrupts service continuity and undermines program performance [3,30,49,53]. Systematic reviews have consistently identified incentives, career ladders, and supportive work environments as critical for CHW retention [111–113]. Addressing this crisis requires a comprehensive retention strategy that includes improved pre‑service and in‑service training, clear job descriptions, task reduction, adequate living facilities at health posts, and a robust incentive system combining financial rewards with career development opportunities.

The HEP’s reliance on community‑level structures such as the Women Development Army (WDA) and model families has weakened over time. WDAs were originally intended as a bridge between HEWs and households, but they became overburdened, inadequately trained, and poorly supervised, leading to burnout, attrition and diminished engagement [12,24,27,32,36,54,62]. Model family training was delivered inconsistently, and trained families often lacked the resources or follow‑up to influence their neighbors [1,39,64]. International evidence confirms that volunteer CHW support structures often falter without sustained investment in training, supervision, and incentives [108,113]. This mirrors experiences in other LMICs where unpaid community volunteers struggle to sustain engagement without systematic support [114,115]. The weak integration of Traditional Birth Attendants (TBAs) further disconnected the formal system from community practices, perpetuating reliance on TBAs for maternal and child health [21,65,66]. Successful TBA integration models in Nigeria show that collaborative approaches, including training TBAs as community allies, linking them to health facilities, can improve service uptake [114]. Negative community attitudes were fueled by the limited-service package, coercive practices (e.g., forcing institutional delivery), and resource constraints (e.g., inability to afford latrine materials) [12,20,22,24,33,43,65,67,68]. These factors mirror findings from other LMICs where CHW programs struggle when they fail to align with community priorities or socio‑economic realities [66,95]. Revitalizing community engagement requires investing in WDA and model families with structured training, small incentives, and regular supervision. Formalizing collaboration with TBAs and ensuring community input into service design can rebuild trust. Addressing resource barriers (e.g., subsidizing latrine materials) would enable households to act on health advice.

At the health system level, the review identified four key barriers undermining HEW performance: fragmented partner engagement, weak supervision, chronic resource shortages, and governance gaps. A lack of harmonized coordination platforms [47,56,66,69,113] for engaging multiple partners (NGOs, government sectors, development programs) with HEWs created conflicting demands and redundant reporting, leaving HEWs overloaded and confused [47,56,66,69]. While fragmentation is common in decentralized health systems, coordinated platforms at the district or PHCU level can reduce duplication and improve efficiency [95,113]. Supervision was frequently described as irregular, unsupportive, or abusive. Performance appraisals were widely viewed as fault-finding rather than constructive [5,7,23,24,30,37,43,44,59,64,70–73,75]. The literature suggests that supportive supervision and regular feedback can enhance CHW performance [70,104]. Resource and infrastructure deficits—such as stock-outs of essential drugs and supplies, lack of office furniture, absence of operational budgets, and poor roads—were reported to severely constrain service delivery and demoralize HEWs [6,7,12,20,24,27,30,40,41,43,47,52,53,76,77,79–82,84–89]. These gaps signal weak governance and what the literature refers to as ‘government apathy,’ defining it as insufficient political commitment and funding [5,12,27,30,35,37,43,47,49,53,66,71,90,92]. These systemic failures are characteristic of decentralized health systems and require sustained political will to address (146, 149). This is consistent with findings from LMIC studies that identify supply chain weaknesses as a key bottleneck to CHW effectiveness [116,117].

The barriers identified across the four themes are deeply interconnected. A design flaw (a preventive‑only focus) influences community attitudes and trust, which, in turn, affects HEW motivation. Weak governance leads to resource shortages and poor supervision, exacerbating workforce turnover and eroding community structures. Therefore, isolated interventions, such as training alone or adding more HEWs without addressing workload, are unlikely to yield sustained performance. A systems approach that simultaneously addresses design, workforce support, community engagement, and governance is needed.

### Limitation of the study

While this review comprehensively maps HEP barriers from program inception to current developments, drawing on a diverse body of literature, several limitations must be acknowledged. As scoping review, prevents quantification of the relative influence of each barrier. In addition, it cannot establish causal relationships between identified barriers and HEP performance outcomes. Publication bias may also affect the findings, since studies with positive or neutral results are less likely to be indexed, and restricting the searches to English-language publications may have overlooked relevant evidence from non-English sources. Moreover as we included many types of studies and grey literature, we did not formally assess the quality of each source. Therefore, our findings provide a descriptive overview rather than a meta-analytic estimate. These limitations underscore the need for future studies to focus on specific contextual barriers to HEP.

## Conclusions

This scoping review mapped multidimensional barriers affecting the HEP and its HEWs, drawing on evidence from 97 documents. The findings showed that program design issues persist. The HEP’s preventive focus does not match the community’s demand for curative services, which weakens trust and acceptance, particularly in urban areas. The female-only HEW workforce, while bneficial for maternal and child health, causes gaps in service continuity and safety, and the performance of physically demanding tasks, and fails to effectively engage men and youth. The HEW workforce faces a crisis of capacity, motivation, and retention. Inadequate pre-service and in-service training leaves HEWs ill-prepared. Excessive workloads, non-healthcare tasks, and CBHI responsibilities contribute to burnout and high turnover. Community engagement and support structures have weakened considerably, with WDA and model family networks overburdened and under-resourced, and the weak integration of TBAs keeps people reliant on informal care. To revitalize these structures, provide structured training and small incentives. Ensure regular supervision, formal TBA collaboration, and community involvement in service design.

At the system level, fragmented partner engagement, ineffective and fault-finding supervision, chronic resource These barriers are deeply interconnected and mutually reinforcing, meaning that disconnected, single-level interventions are unlikely to succeed.

### Implications

Addressing these barriers requires a holistic, systems-oriented revitalization of the HEP that acts simultaneously across program design, workforce, community, and governance levels. At the program design level, integrating select curative services or strengthening referral pathways would better align the HEP with community expectations, while recruiting male HEWs would create a more balanced, resilient, and inclusive workforce capable of engaging men and youth.

For the workforce, a comprehensive retention strategy is essential. This should encompass competitive salaries, career ladders, and recognition mechanisms, alongside improved living conditions at health posts and a reduction of non-health tasks. CBHI duties should be formally reassigned to dedicated personnel to relieve HEWs of administrative burdens that fall outside their core mandate. Pre-service and in-service training must also be strengthened to ensure HEWs are adequately prepared for the full scope of their responsibilities.

To revitalize community engagement, structured training and small incentives should be provided to WDA and model family networks, supported by regular supervision. Formal TBA collaboration should be institutionalized to bridge informal and formal care systems, and communities should be meaningfully involved in service design and performance appraisal to rebuild trust and ownership of the program.

At the system level, district-level coordination platforms are needed to harmonize partner engagement and reduce redundant demands on HEWs. Supervision must shift from irregular and fault-finding practices toward supportive models that provide constructive feedback and foster motivation. Ensuring a reliable supply chain, addressing infrastructure deficits, and securing sustained political commitment and adequate funding from local to national levels are all prerequisites for a functional and accountable enabling environment. Finally, future research should focus on implementation science and quantitative studies to evaluate the impact of targeted interventions and identify the most effective strategies for optimizing HEP performance across Ethiopia’s diverse contexts.

## Supporting information

S1 File Search Strategy (PubMed). (DOCX)

S2 File Barrier grand summary table. (DOCX)

S3 File Preferred Reporting Items for Systematic reviews and Meta-Analyses extension for Scoping Reviews (PRISMA-ScR) Checklist. (DOCX)
