# Supplementary material for: Mapping multilevel barriers impacting the performance of Ethiopia’s Community-Based Health Extension Program: A scoping review
Source: PLoS One. 2026 Jul 20;21(7):e0351989. doi: 10.1371/journal.pone.0351989 (PMC13384300; doi:10.1371/journal.pone.0351989)
Supplement: S2 File — (DOCX) [file pone.0351989.s002.docx]

**Supporting Information II: Barrier Grand Summary Table**

| **P** | **Authors** | **Year** | **Context (Urban, Rural, Pastoral, Mixed)** | **Setting ( facility, community based …** | **Regions (e.g., Amhara, Oromia)** | **Type of Source/Document (e.g., Published, Grey Thesis, Government Reports)** | **Study Aim/ Objective** | **Focus of Study/Report (e.g., Satisfaction)-HEP component such as MCH, EPI, sanitations etc** | **Designs and Methods (Key Design Elements)** | **Population Addressed** | **Barriers, Challenges** |
| --- | --- | --- | --- | --- | --- | --- | --- | --- | --- | --- | --- |
| P1 | CNHD, C. f. N. H. D. i. E | 2007 | Rural | community based | Oromia, Amhara | Grey | Monitor the HEP performance in the provision of the health service packages, and contribute information for the evaluation of the impact of HEP | Performance | Experimental | Households/Villages | Lack of adequate accommodation at health posts, low salaries relative to workload, limited access to transportation, inadequate training, unclear job descriptions upon deployment, low participation community in HEP activities, and limited opportunities for career advancement, and transfers or promotions. In addition, they reported excessive workload and associated stress, and burnout. The limited scope of HEP or lack of curative or clinical services, inadequate resource allocation, and insufficient vaccine and medical supplies, as well as poor infrastructure such as road networks, further compound the challenges. Socio-cultural factors, such as gender roles, community acceptance, and communication barriers influence HEW performance. A lack of supervision support, and refresher training combined with excessive administrative burdens budgetary constraints, and hostile work environment resulted in demotivation among HEWs |
| P2 | FMoH | 2013 | Not stated | NA (strategic plan) | Oromia,SNNPR, Ahmara, Harari, Gambella, DD, BG,Afar, Addis Ababa | Grey | To review the annual performance of HSDP | HEP performance | Mid-Term Review HSDP IV Regional Reports | Not stated | High HEW attrition, limited career development , absence of office space, insufficient budget, limited technical skills among HEW, few HEWs per kebele, weak supervision, discouraging certification processes low community awareness, poor documentation, inadequate political commitment lack of transportation, scattered communities, a lack medical equipment and supplies, community mistrust in HEWs and their diversion to non-health tasks such being enforced to be election board member or otherwise under the threat of salary cuts, impede HEP performance |
| P3 | Higi, A. H., et al | 2021 | Rural | Facility based | Oromia, Amhara, and SNNNP | Published article | Explore the perception and experience of HEWs toward facilitators and barriers to maternal and new-born health care seeking and utilization. | Maternal and new-born health service | A descriptive qualitative study | HEWs | Financial constraints, prioritization of home births by families and mothers-in-law due to cultural beliefs, the preference for home births stemming from positive past experiences, the imposition of fees for maternal services, Geographic barriers, such as poor infrastructure and transportation, a lack of spousal support, inadequate medical supplies, prevailing cultural and religious practices surrounding childbirth impended l maternal healthcare services use. |
| P4 | Alemu, T. | 2014 | Rural | Community based | Oromia | Thesis | To assess communities’ perception towards the program | Implementation of Health extension service | A community based cross-sectional study design utilizing both quantitative and qualitative data | HEWs, women lead health development army leaders, supervisors of health extension program, and the woreda health office head | Inadequate infrastructure, ill-equipped health posts and a shortage of essential supplies, Weak collaboration across sectors, un aligned of HEP activities with other developmental initiatives, and ambiguity regarding the roles of HEWs with other stakeholders hinder the HEP performance. limited training participation of the model family due to time constraints, disinterest, and geographical barriers further hinder progress. Lack of support from local leaders, community resistance, insufficient sanitation facilities, and inadequate supervisory assistance poses additional barrier |
| P5 | Gadisa, S. | 2010 | Not stated | Not stated | Oromia | Thesis | Assess Health Extension Workers (HEWs) functionality and competence on Family Health Package components of Health Extension Program | Functionality and competence on family health package | Descriptive cross sectional study design | HEWs | Insufficient supportive supervision, insufficient refresher training, lack of reference materials at health post, inadequate recommended medical equipment, and lack of living house at the health at health post. Pre-service training is inadequate as most HEW feel ill-equipped to carry out HEP activities. The practice setting at the health centre is unsuitable due to the absence of a demonstration room, library, and adequate staffing |
| P6 | Gebretsadik, A., et al. | 2020 | Not stated | Not stated | Sidama | Published article | Exploring community acceptance and utilization of the CBNC services | Maternal and community-based neonatal care services | a qualitative study | Mothers, HEWs, and HEW Supervisors and Coordinators. | Persistent absence of HEWs at health posts, a prevalent misunderstanding within the community, HEWs lack crucial skills related to diagnosing and managing neonatal illnesses, Community resistance, and inadequately equipped health posts. The demotivation among HEWs, overwhelming workloads, and lack of essential resources such as transportation, manpower, and budget allocations. The absence of consistent monitoring and evaluation by the District Health Office and Primary Health Care Units, insufficient supplies at health posts, inadequate infrastructure, including water and electricity supply |
| P7 | Arora, N., et al. | 2020 | Mixed | Facility based | SNNPR, Tigray | Published Article | Identify factors influencing the retention of CHWs in Ethiopia | Retention of CHWs | A systematic review | HEWs, leavers and policymakers | The discrepancy between the salaries of HEWs and the high workload they bear and meeting living expenses. Turnover among HEWs resulting from conflicts with supervisors and senior managers. lack of effective leadership within the sector. Low Community acceptance of preventive health activities. The lack of adequate medical supplies. competing interests HEWs face between their professional responsibilities and family obligations, especially when their families reside in different locations from their work place. safety concerns for HEWs traveling to rural areas for health outreach activities. The lack of opportunities for HEWs to transfer limits career development within the sector. Demotivation stemming from a lack of support, recognition, and rewards from supervisors’ underscores. |
| P8 | Birhanu, Z., et al. | 2013 | Rural | community based | Oromia | Published Article | Assess mothers’ experiences and satisfactions with HEP | Mothers’ experiences and satisfaction | Community based cross sectional study | women of reproductive age group | Absence of curative health services at health posts, limited male participation, gender partiality in HEP, safety concern of HEW for traveling at night to assist women in labour, two HEW is not sufficient per health post, and lack of acceptance and trust from certain community members towards HEWs. |
| P9 | Amare, Y., et al. | 2018 | Rural | community based | Ahmara, SNNPR | Published article | Exploring barriers and facilitators to timely postnatal visits t with families and Health Extension Workers in four Ethiopian sites. | Early postnatal home visits | Qualitative study | mothers, grandmothers, fathers, HEWs and HDA members | Geographical barrier, HEW unavailability at health post and heavy workloads and unavailability hinders HEP performance |
| P10 | Adamu, H. | 2012 | Urban | Mixed(Facility and community based) | Oromia Regional State | Thesis | Assess factors that affects the implementation of urban health extension program | Implementation of urban health extension program | cross-sectional design with qualitative methods | urban health extension workers (UHEW) and urban health extension supervisors (UHES), kebele representatives, women representatives and functional public health centers | Community resistance, budget limitations, high turnover of HEWs, weak relationships between workers and health centers, and a shortage of first aid materials hinder HEP |
| P11 | Zebre, G., et al. | 2021 | Urban | Mixed(Facility and Community based) | Addis Ababa | Published Article | Exploring the implementation, strategies, experiences and challenges for the successful implementation of UHEP in Addis Ababa | Implementation, experience, and challenges of the urban HEP. | A qualitative case study | urban HEP, urban HEP supervisors, Health Development Army leaders, HEP administrators, and other community members | Inadequate materials and equipment, insufficient training duration, conflicting directives, lack of supervision, geographical obstacles, shortages of essential supplies, heavy workloads, low salaries, limited career development opportunities, and managing additional tasks from different stakeholder without a clear framework. |
| P12 | Gebreegziabher, E. A., et al. | 2017 | Urban | Community based | Oromia Regional state | Published Article | Exploring utilization of urban health extension services | Accepted and use of Urban health extension services | A community based, descriptive, cross sectional study, used both quantitative and qualitative methods | Urban Health Extension Professionals, kebele health committee members and Households | Limited availability of time among urban dwellers and resistance from the community to engage in HEP initiatives |
| P13 | Daka, D. W., et al. | 2023 | Rural | Facility based | Oromia, Amhara, Tigray and Southern Nations | Published Article | Examining association between quality of assessment, classifcation and treatment of sick underfve children by health extension workers at health posts | Quality of sick child management | Baseline and end-line surveys in the intervention and  comparison | sick children 2–59 months old | Lack of continuous mentoring and support |
| P14 | Kassa, H. | 2019 | Rural | Facility Based | Amhara Region | Thesis | Assess health extension workers’ medicine supply chain management practice and associated challenges in selected primary health care units | Medicine Supply Chain Management Practice | A qualitative study using in-depth and key informant individual interview, focus group discussion (FGD) and observation | HEWs, Pharmacy professionals, and female community members | Shortage of essential medicine supplies and the lack of adequate transportation between health centres and health posts. HEW overloaded by tasks unrelated to HEP, such as registering community for health insurance and distributing insurance ID cards to households. Insufficient training in managing medical supplies and medicines, including shelf life and inventory management. disparity between the workload of HEWs and their salaries. Lack of recognition and reward for their efforts. Supervisor demoralize HEWs by focusing on minor issues during supervision instead of recognizing and rewarding their efforts. |
| P15 | Bekele, A., et al. | 2008 | Rural | Mixed(facility and community based) | SNNPR | Published Article | Generate useful information on the implementation of the newly initiated health service extension program | Implementation of the health services extension program | Cross sectional - survey, health impact evaluation survey, both  quantitative and qualitative methods | HEWs | Only two HEWs per village is inadequate to carry out HEP activities. high HEW turnover rates, lack of a clear career path for HEWs leads to job dissatisfaction. Unattractive salary scale for woreda HSEP coordinators. Shortage of medical equipment and supplies. Lack of budget for HEP activities. Absence of a well-established referral system. Limited supportive supervision |
| P16 | FMoH | 2018 | Mixed | Mixed,Sytem level | National , Ethiopia | Grey | Highlights progress, Challenges, lessons learned and Way Forwards for the health sector for the forthcoming year | HEP performance | Annual Health Sector Performance | HMIS/PHEM, CSA, application of accountability scorecard, Central Statistical Agency (CSA) | Low commitment of the leadership at all levels towards the health extension package. Weak WDA structure, functionality, and participation in HEP. Lack of attitude and uniformity in institutionalizing WDAs. Weak inter-sectoral collaboration and institutional support. Ineffective follow-up and monitoring system |
| P17 | Feyissa, A. 2011 | 2011 | Rural | Facility based | Oromia Regional State | Thesis | Determining the magnitude of attrition among Health Extension Workers deployed and explore the whereabouts of Health Extension Workers who have left their jobs and perceived reasons related to it | Attrition, explore the whereabouts of the HEWs who have left their jobs | An Exploratory Cross-Sectional study, having document reviewing | HEWs | Low salary limited opportunities for career advancement, and overwhelming workload poor working and living condition due to inadequate accommodation, furniture, and medical supplies. lack of recognition, rewards, and incentives for hard work demotivated HEWs transfer to a different location and o family problems as they work far from their children and spouse |
| P18 | FMoH | 2008 | Mixed | Mixed (Facility and community based) | National , Ethiopia | Grey | Measure and document the extent to which the targets set for the HSDP III are achieved, assess constraints and/or challenges encountered and solutions provided, draw best lessons learned and experiences gained to improve future management and implementation of activities to attain the HSDP III goals |  | Mid-Term Review: The qualitative and quantitative questions, served as a guide during the interviews | KII (Governmental & NGOs) and documemnent review HSDP I & HSDP II | Limited health post, transportation facilities and Lack facilities for HEW at Health Post. HEW withdraw from training and unwillingness to stay in a rural area, HEW turnover and HEWs on maternity leave compromised health posts functionality. |
| P19 | Befekadu, A. and M. J. B. P. H. Yitayal | 2020 | Rural | Facility based | West Gojjam zone, Amhara Region | Published Article | Assessing the HEWs’ knowledge and practice on drug provision for childhood illnesses and factors associated with it | Drug provision for childhood illness | Institutional based cross-sectional study | rural HEWs | Absence of national guidelines at health posts |
| P20 | Workie, N. W. and G. N. Ramana | 2013 | NA | Not stated | Amahra; Oromia; Southern Nations, Nationality and Peoples; and Tigray regions. | Grey | Assess the contribution of HEP on health outcome of households and community | The context for the introduction of the program The scope of the service package delivered under the program;  The institutional arrangements and the links with the rest of the health system | Case study | Not stated | HEWs not provided with sufficient practical training to identify danger signs and assist a woman in labour |
| P21 | Shuke, A. | 2014 | Urban | Facility based | Sidama Zone, Southern Ethiopia | Thesis | Assess the knowledge and practice of HEW regarding the medicine they handle | Knowledge and Practice of Medicine they Handle | A cross-sectional descriptive study | HEWs | Certain health posts either lack refrigeration or have non-functional ones. stockouts of essential medications like chloroquine, Coartem, ORS, and family planning pills. |
| P22 | FMoH | 2019 | NA | System level | National , Ethiopia | Grey | Generating lessons that can be used to advance the implementation of the country wide Woreda Transformation initiative |  | Annual report | Not stated | Lack of knowledge on part of community about the availability comprehensive family planning, weak integration between the health centers and urban health extension professionals. weak collaboration and accountability among sector ministries on HEP. High turnover of health managers and officers from regional to Woreda level. Weak documentation and reporting |
| P23 | Dereje, Y. | 2022 | Mixed | Facility based | Amhara Region | Thesis | Asses data management practice and its associated factors among health extension workers | Data Management Practice | Facility based cross- sectional quantitative study supported with qualitative approach | Health extension workers | Lack of knowledge among HEW on quality data management and CHIS due to lack of training and CHIS formats not friendly and written English which is difficult for HEW. HEWs fabricate and inflate data as the district cadre appreciate those who report higher false report than real one. Lack of necessary input like updated family folder that matches with population growth, standard registration, tally sheet, integrated card to implement CHIS. Lack of reward and recognition both from community and the officials |
| P24 | FMoH | 2016 | Mixed | Sytem level | National , Ethiopia | Grey | Highlighting the three key features of quality and equity, universal health coverage (UHC), and transformation under four pillars of excellence of HSTP: (i) Health Service Delivery; (ii) Quality Improvement and Assurance; (iii) Leadership and Governance; and (iv) Health System Capacity |  | Annual performance report | Health Management Information System (HMIS)/PHEM aggregated monthly and quarterly and annual reports and CSA | Weak collaboration, commitment and skill among sector offices to implement support HDA. Lack of regular supporting supervision Inadequate performance of graduated Model Households on HEP some packages. |
| P25 | Sibamo, E. L. and T. M. J. B. h. s. r. Berheto | 2015 | Rural | Community based | Southern nations and nationalities regional state | Published article | Provide insight into the overall satisfaction of the community towards UHEP and the services provided by UHEWs | Community satisfaction with the urban health extension service | cross-sectional study | households, village health committee members and model families | Some HEWs are dissatisfied |
| P26 | Wang, H., et al. | 2016 | NS | Mixed | National | Grey | Ethiopia health extension program: an institutionalized community approach for universal health coverage | UHC | A quantitative analysis | Two Ethiopia Demographic and Health Surveys (EDHSs) | Lack of uniformity between region on the proportion of model |
| P27 | Hussien, M., et al. | 2020 | Rural | Facility based | Amhara Region | Published Article | Assess the magnitude of HEWs’ intention to leave their jobs and factors associated with their intention | Intention to leave their jobs | cross-sectional study | HEWs | HEWs Intention to their job due to inadequate salary, high workload, dissatisfaction reward mechanisms and poor working environments |
| P28 | Tekle, M. G., et al. | 2022 | Rural | Mixed | Addis Ababa, and Dire Dawa | Published Article | Assess the magnitude of, and factors associated with the attrition and intention to leave of HEWs currently working as part of the heath system | Attrition and intention to leave | a cross-sectional survey | HEWs | HEW turnover due to heavy workload, influence from family, health issues, safety concerns, limited skills, conflicts with authorities, lack of community acceptance, time constraints for childcare, ineffective performance evaluations, denial of rightful leave, unresponsive management, discrimination, lack of respect, inadequate supervision, and failure to fulfil promises. Additionally, insufficient incentives like low pay, unappealing incentive schemes, absence of acknowledgment, and limited career prospects can also contribute to turnover |
| P29 | Daka, D. W., et al. | 2020 | Rural | Facility based | Oromia, Amhara, Southern Nations, Nationalities, and Peoples (SNNP) and Tigray | Published Article | Aassessing the quality ofthe health care provided to sick children mobilized to visit health posts. Specifically, we examined the assessment, classification,treatment, counseling and referral done by the health extension workers at health posts in four regions ofEthiopia. | Quality of clinical assessment and management of sick children | A facility-based cross-sectional study was carried out involving observation of clinical consultations and interviews. | children 2 to 59 months | low community awareness, unfavorable perceptions of care quality, and inadequate practices by HEWs |
| P30 | Defar, A., et al. | 2021 | Rural | Community based | Dangla and Ankesha, of the Awi zone in the Amhara regional state | Published Article | Exploring mothers’ and health extension workers’ perceptions and experiences of the outreach services provided for the management of childhood illnesses | Outreach Management of Childhood illnesses | A qualitative study | health extension workers and mothers or other caregivers of under-ﬁve children | Less at home support from HEW when children are sick. lack of trust in the health extension workers’ skills and the lack of diagnostic tools, essential drugs during home visits, lack of effective communication to call them children got sick. Lack of curative service at health post. lack of commitment on part of HEWs. Lack of transportation with in the village |
| P31 | Mangham-Jefferies, L., et al. | 2014 | Rural | Community-based | Southern Nations Nationalities and People’s Region and Oromia Region | Published article | Evaluate a package of community-based interventions for newborns. | Time allocation | Experimental | HEWs and community health volunteers | HEWs effort and time management on different HEP activities |
| P32 | FMoH | 2020 | NA | NA (Strategic plan) | National , Ethiopia | Grey | Review HEP performance |  | Ethiopian Health Sector Transformation Plan II | PHCUs (Health posts & health centres) | Lack of essential resources such as water, electricity, phone services, and sanitation. HEWs face challenges including high turnover rates, low morale, and inadequate skills. Male and youth HEWs are particularly marginalized in HEP package. The capacity of HEWs is limited, and local governance structures, like the WDA, are underperforming due to factors such as unclear selection processes, a lack of strong leadership, and excessive reliance on the WDA framework. |
| P33 | Mamo, A., et al. | 2019 | Mixed | Community Based | Oromia | Published Article | Exploring the role played by different actors in promoting ANC, childbirth and early PNC services, and mainly designed to inform a community based Information, Education & Communication intervention | Roles of community health actors in promoting maternal health services | An exploratory qualitative study in-depth interviews and focus group discussions | HEWs, religious leaders, Women Developmental Army leaders (WDA), Male Developmental Army leaders (MDA) and married male and female community members | Community poor attendance at community meetings, un engaged men/husbands, and religious influence affect HEP service utilization |
| P34 | Abate, M., et al. | 2022 | Mixed | Facility based | Amhara, Oromia, South nations, and nationalities people’s region (SNNPR) and Tigray | Published Article | Determine the extent and variation of health professionals’ motivation alongside factors associated with motivation | Factors influencing motivation | facility based cross-sectional study | nurses and midwives, HEWs and staff representing case team leaders, facility and district heads, directors, and officers | Heavy workload demotivates HEWS |
| P35 | Lifson, A. R., et al. | 2018 | Rural | Community Based | the Southern Nations Nationalities and Peoples Region (SNNPR) of Ethiopia | Published Article | Assess an intervention using CHWs to improve retention in HIV care | counselling and support for patients newly entering HIV care | RCT: A Multi-Site Community Randomized Trial of Community Health  Workers | Adults newly entering HIV care and CHW | Resistance from the community towards home education practices by HEWs. Far distance from health post, lack of phones, and community mobility hinder HEP service |
| P36 | Jackson, R., et al. | 2016 | Rural | Mixed (Facility and community based) | Tigray Region | Published Article | Exploring HEWs and mothers’ attitudes to maternal health service utilization and acceptance using a peer informant approach. | Attitudes to maternal health service utilization and acceptance | Key Informant Monitoring and Participatory Ethnographic Evaluation and Research | HEWs or women | Socio-cultural factors, such as limited transportation, traditional beliefs like *zwar*, and patriarchal decision-making within families (grandmothers, or mothers-in-law), hinder HEP service use at health facilities and for example pregnant to give birth at home with help of traditional birth attendants. Poor quality of service of at health facilities, and previous good home delivery experience pose additional hinderance |
| P37 | Afework, M. F., et al. | 2014 | Mixed | Community based | Tigray and Southern Nations, Nationalities, and People’s Region (SNNPR) | Published Article | Assess the role of Health Extension Workers in improving women’s utilization of antenatal care, delivery at health facility and postnatal care services | Maternal health service | community based cross sectional study | all women 15–49 of age, married or unmarried | Inadequate home visit by HEW |
| P38 | Admassie, A., et al. | 2009 | Rural | Mixed | Amhara, Oromia, and Southern Nations, Nationalities and Peoples Region | Published Article | Evaluate the short-term and intermediate-term impacts of the programme on child andmaternal health indicators in the programme villages | Impacts of HE programme on child and maternal health indicators | Comparative study | Villages, households and HEWs | Geographical inaccessibility limits access to healthcare for rural communities |
| P39 | Fetene, N., et al | 2016 | Mixed | Mixed | Oromia, Amhara, Southern Nations, Nationalities, and Peoples’ Region (SNNPR) and Tigray, Addis Ababa | Published Article | Assessing variation in the implementation of the primary health care efforts | Variation in the implementation of the primary health care | qualitative methods | Administrative staff and health development armies | Limited and fragmented support from health centers and the community hindered WDA development. Additionally, HEW demotivation due to perceived career stagnation, financial compensation for high workload, unclear role description, non-supportive supervision, poor community relationships, and the absence of curative services within HEP packages further affects HEP performance. Urban residents prioritize physician-led primary care as the view that UHEP less qualified. A top-down reporting culture, bypassing health center focal persons, hinders evidence-based problem-solving and collaboration. Mismatch between HEW role and community expectation of curative service that resulted in community resistance. Urban settings pose unique challenges for HEWs due to the diverse population, varied lifestyles, and irregular work schedules, making it difficult to reach residents at home |
| P40 | Gela, B. D., et al. | 2014 | Rural | Community based | Oromia | Published Article | Determine prevalence of ANC utilizations and  associated factors from health extension workers | ANC utilization | Community based cross sectional study with systematic random sampling | women | Geographical inaccessibility of health facilities reduces community participation in HEP services. |
| P41 | Yahya, M. | 2011 | Mixed | Facility based | Oromia National Regional State | Thesis | Determine the factors that contribute to motivation and attrition of HEWs | HEWs motivation | Cross sectional study design using both quantitative and qualitative data | HEWs, Health Extension Supervisors, Health Extension Package coordinators | HEW dissatisfied due to lack of unattractive payment, community support, enough time for personal activities, career development opportunities and motivation |
| P42 | Gebretnsae, H., et al. | 2022 | Rural | Mixed | Tigray region | Published Article | Assessing the contribution of HEWs on TB case notification and its associated factors | TB case notification | concurrent mixed method (quantitative and qualitative) cross-sectional study | HEWs | high workload of HEW, and the financial burden associated with transportation |
| P43 | Medhanyie, A., et al. | 2012 | Rural | Community based | Tigray region | Published Article |  | Performance of HEWs of Maternal health | a cross-sectional study | HEWs | Lack of appropriate protocols and aids at health post for HEP service implementation |
| P44 | Molla, S., et al. | 2020 | Urban | Community Based | Amhara | Published Article | Assessing the level of women satisfaction with urban HEP | Women satisfaction with HEWs perfomance | A community-based cross-sectional study design | Women aged 18 years and older | Unavailability of HEW at health post in most of the time |
| P45 | Shaw, B., et al. | 2016 | Rural | Community based | Rural zones of Jimma and West Hararghe | Published Article | Elicit perceptions and experiences of caregivers to better understand reasons for low utilization of iCCM services | integrated community case management of childhood illnesses | A qualitative study of the perspectives and experiences of caregivers | mothers of children under the age of five, HEWs and members of the HDA (including VCHWs), women’s husbands | The frequent absence of HEWs at health posts, geographic challenges, and poor transport options hinder access to remote areas. Despite free services, financial barriers include indirect costs like transportation. Negative perceptions of HEW interactions and concerns about medicine quality reduce utilization rates |
| P46 | Medhanyie, A., et al. | 2012 | Rural | Community based | Tigray region | Published Article | Assess utilization to maternal health services by women in rural villages in Ethiopia | Maternal health service | a cross sectional study | Women with under-five children | inadequate access to health facilities, underutilized health posts due to poor infrastructure, and lack of essential supplies. Cultural beliefs favoring home births, knowledge limitation among HEWs, illiteracy of the service users |
| P47 | Bayou, N. B. and Y. H. M. J. E. j. o. h. s. Gacho | 2013 | Rural | Community Based | SNNPR | Published Article | Determining the utilization of clean and safe delivery service and identify the factors influencing the utilization | Maternal health service | a community based cross-sectional survey | Women | Unavailability of HEW at a health post most of the time. Community not confident on HEWs ability |
| P48 | Miller, N. P., et al. | 2021 | NA | NA(Review) | Not stated | Published Aricle | Assess barriers to the utilization of HEW services and to explore potential solutions | child and newborn health services | a scoping review | peer-reviewed articles and grey literature | Community-level barriers to healthcare access include limited knowledge of illness danger signs, financial constraints, and transportation challenges. System-level barriers comprise drug shortages, provider disrespect, inadequate HEW skills, and low provider confidence |
| P49 | Negussie, A. and G. J. B. H. S. R. Girma | 2017 | Rural | Community based | SNNPRS (Southern Nations, Nationalities and Peoples Regional State) | Published Article | Assess the role of HEWs in MCH service delivery | MCH | A community based cross-sectional | Mothers with under-five children | Inadequate training and support for HEWs, low community awareness of services, and insufficient home visits. Cultural beliefs, distance to health posts, and lack of transportation complicate access |
| P50 | Kane, S., et al. | 2016 | NA | Systematic Review | A multi-country study | Published Article | Examining the ‘empowerment experience’ of CHWs as part of the REACHOUT study which is being conducted in Ethiopia, Malawi, Mozambique, Kenya, Bangladesh and Indonesia; | Community health worker empowerment | a multi-country comparative study | Documents (policies, practices, articles) | Unconstructive supervision as some supervisors is fault finder |
| P51 | FMoH | ????? | NS |  | National , Ethiopia | Grey | Establish an eCHIS in Ethiopia is to contribute to achieving  the goal of universal coverage of primary health care in Ethiopia, in both agrarian, urban and pastoralist areas of the country |  | Information revolution | Not stated | High workload of HEWs, shortage of CHIS tools, Limited supportive supervision to the health post from health center and woreda health office, and high turnover of trained HEW |
| P52 | Gilano, G., et al. | 2017 | Rural | Community based | Southern Nations Nationalities and Peoples Region (SNNPR) of Ethiopia. | Published Article | Determine factors associated to information sharing among  the health extension workers. | information sharing | A cross sectional study | HEWs | Poor information management? |
| P53 | Tadesse, A., et al. | 2022 | Urban | Community based | Amhara Region | Published Article | Assess the impact of HEP on diarrheal disease among under-fve children and to identify factors associated with diarrheal disease | Diarrheal disease among U5 | A community-based cross-sectional study design | under-five children | Fewer home visits linked to increased childhood illness. |
| P54 | FMoH | 2010 | NA | NA(Strategic plan) | National , Ethiopia | Grey | Review on HEP performance |  | Health Sector Development Program IV | Not stated | Limited HEW career growth, poor training coordination, unavailability of HEWs on weekend/night time at health post, low community ownership, ineffective model household training, and overall capacity constraints. |
| P55 | Getachew, T., et al. | 2021 | Rural | Facility based | Amhara; Tigray; Oromia; Southern Nations, Nationalities, and Peoples | Published Article | Assess the health extension workers’ perceived  health system context and the health posts’ service readiness in four Ethiopian regions. | Context and health post preparedness | Interventional study | HEWs | lack of adequate staffing, communication tools, essential medicines, and clinical guidelines. Inadequate community engagement and continuous learning opportunity |
| P56 | Yitayal, M., et al. | 2014 | Rural | Community based | Amhara Region | Published Article | Assessing the utilization of the HEP by the community and factors that might be associated with it. | Household visit | community-based cross-sectional survey | Women (RAGs) | Fewer home visit by HEW linked with less HEP service use |
| P57 | Tamene, A., et al. | 2023 | Mixed | Facility based | Southern Nations Nationalities and Peoples  Region (SNNPR). Hadiya | Published Article | Assess the job satisfaction of HEWs and associated factors | job satisfaction | A facility-based cross-sectional study | rural health extension workers | Inadequate compensation and incentives, poor infrastructure, limited support and supervision, lack of recognition for HEWs, insufficient training opportunities, high workloads leading to burnout, and logistical challenges due to long travel distances to health posts |
| P58 | Kok, M. C., et al. | 2015 | Rural | Community Based | Sidama zone of the South Nation Nationalities and Peoples Region of Ethiopia | Published Article | To assess relationships between HEWs, the community and health sector were shaped, in order to inform policy on optimizing HEW performance in providing maternal health services | Quality assessment | Qualitative study, FGDs and semi-structured interviews | HEWs, TBAs, health professionals and community members | Miscommunication and lack of referral forms, Supervision from health sector authorities was often perceived as fault-finding rather than supportive, Inadequate Training Opportunities, Irregular Monitoring and Evaluation, disrespect or lack of support from their supervisors, Expectations Misalignment, |
| P59 | Mathewos, B., et al. | 2017 | Rural | Community based | National , Ethiopia | Published Article | Assess whether the programme was cost-effective and affordable | maternal and newborn service | Community-Based Interventions for Newborns in Ethiopia (COMBINE): Cost-effectiveness analysis | kebeles in the intervention area and HEWs | Lack of resource |
| P60 | Gebretnsae, H., et al. | 2020 | Rural | Facility based | Tigray region | Published Article | Assesing the implementation status and associated factors of HH contact screening by HEWs | TB screening | programme assessment | All health facilities (primary hospitals and health centers) | Inadequate household visits by HEWs), limited health education for households, restricted access to public transport and long distances to health facilities, and insufficient collaboration between HEWs and community structures like the WDA |
| P61 | Ameha, A., et al. | 2013 | Rural | Facility based | 113 districts of Ethiopia | Published Article | Assess the effectiveness of visits to improve the consistency of iCCM skills (CoS) of the HEWs in 113 districts in Ethiopia | Suportive supervision | longitudinal secondary anal ysis of program monitoring | health posts | Challenges related to the quality of training and ongoing support for HEWs. Insufficient resources and inadequate supervision, the high turnover of health workers, and limited access to necessary medical supplies |
| P62 | Jackson, R. and A. J. E. j. o. h. s. Hailemariam | 2016 | Mixed | Mixed | Afar Region, Southern Nations, Nationalities and Peoples’ Region (SNNPR) and Tigray Region. | Published Article | Exploring what pregnant women and HEWs view as barriers to and facilitators for SBA, and for referral to midlevel health facilities for birth | maternal health/linking pregnant women | Qualitative study | HEWs, women and staff from regional, zonal, and woreda health offices and hospitals | Limited maternal autonomy in scheduling ANC visits, inadequate waiting areas, and fear of C-sections deterring early childbirth facility attendance. |
| P63 | Kim, S. S., et al. | 2015 | Mixed | Mixed | SNNPR and Tigray | Published Article | Assess implementation fidelity along the continuum of the delivery process of a community-based IYCF intervention in Ethiopia, to inform program progress toward impact | infant and young child feeding | larger process evaluation (PE) | HEW supervisors, HEWs, community volunteers, and beneficiaries | Low levels of awareness among community members |
| P64 | Datiko, D. G., et al. | 2017 | Rural | Community based | Sidama Zone, in the Southern Nations, Nationalities, and Peoples’ Region (SNNPR) of Ethiopia. | Published Article | To determine the role of HEWS in TB case finding and treatment | TB case detection and treatment outcome | community-based intervention | Individuals with TB and Health centers | Poor accessibility of services |
| P65 | Abeshu, A. | 2017 | Rural | Community Based | Benishangul Gumuz regional state | Thesis | Assessing community satisfactions and associated factors with health extension program | Community satisfaction with HEP | A community based cross-sectional study, using quantitative and qualitative methods | selected mothers and head of the households | Inconsistent HEW availability, infrequent home visits, and minimal community participation impede HEP service utilization |
| P66 | Rudgard, W. E., et al. | 2022 | Mixed | Mixed | Amhara; Oromia; Southern Nations, Nationalities, and Peoples’; and Tigray | Published Article | Investigate the association between household support from HEP and 12 indicators of adolescent health and wellbeing. | Adolescent health service and wellbeing | A Quasi-Experimental Study | Children | Limited Resources (funding) |
| P67 | FMoH | 2016 | NA | NA(Strategic plan) | National , Ethiopia | Grey | Annual performance report | Health Sector Transformation Plan I | Annual report | Not stated | Regional disparities on the Implementation HDA, managerial capacity gaps, suboptimal performance of model households, insufficient supervisory staff, and poor inter-sectoral collaboration |
| P68 | Maes, K., et al. | 2013 | Urban | Community based | Ethiopia and Mozambique | Published Article | Assess the intention behind becoming community health worker | retention of HEWs | Ethionographic study | CHWs | Low or inconsistent compensation, ongoing economic challenges, equality, inadequate Support and Resources, recruitment and retention of CHW |
| P69 | Mengistu, B., et al. | 2019 | Rural | Facility based | Amhara Region | Published Article | Assess the data management practice of HEWs | Primary healthcare data | institution based cross-sectional study | HEWs | Poor knowledge data management practice among HEW due to lack of reference material and training |
| P70 | Getachew, T., et al. | 2021 | Rural | Facility based | Amhara, Tigray, Oromia, Southern Nations, Nationalities and Peoples of Ethiopia | Published Article | Evaluating the ability of HEWs to correct reclassify common early childhood illnesses | common childhood illnesses | Pragmatic design | sick children from 2 to 59 months | Limited competences to classify childhood illness |
| P71 | Dynes, M. M., et al. | 2015 | Rural | Facility based | West Gojam Zone, Amhara region | Published Article | Determining the influence of willingness and ablity of diverse community based MNH workers | MCH workers work interactions (‘teamwork’) | Social network data was collected via purposive snowball sampling strategy. | community health workers | Resource constraints and HEW capacity limitations hinder effective infant care. |
| P72 | Marsh, D. R., et al. | 2014 | Rural | Community based | National , Ethiopia | Published Article | Characterize how HEWs spend their time | INTEGRATED COMMUNITY-BASED CASE MANAGEMENT | review of data related to iCCM program inputs and processes | HEWs and a volunteer Community Health Promoter | Time management capacity limitation |
| P73 | San Sebastian, M. and H. Lemma | 2010 | Rural | Mixed | Tigray | Published article | Estimate the technical efficiency of a sample of health posts | Efficiency of the health extension programme | a data envelopment analysis. | rural districts | Inefficient health posts, lack of guidelines, and weak health management information systems hinder effective implementation |
| P74 | FMoH | 2020 | Mixed | System Level | National , Ethiopia | Grey | Highlighting progress of the different programs, challenges and way forward for the health sector in the forthcoming years | Annual Performance Report | Analysis of both routine and population based data sources | Regional Health Bureaus, the different directorates of the MOH, and development partners | Changes in demography, disease epidemiology, socioeconomic factors, community demand, and global and national affect HEP performance.  Weak WDA engagement in HEP package implementation. Lack detail operational plan |
| P75 | Shaw, B., et al. | 2015 | Rural | community based | Oromia | Published Article | Assess knoweledge gap in ICCM service among community | integrated community case management of childhood illnesses | A cross sectional survey | Child’s primary caregiver | Low caregiver awareness of available treatments, geographical distance from health posts, and perceptions of illness severity and service quality |
| P76 | Aynalem, B. Y. and M. F. J. P. O. Melesse | 2021 | Rural | Community Based | Amhara Region | Published Article | Assess health extension package utilization and associated factors | Health extension service utilization | A community-based mixed cross-sectional study | Households | WDA lacks knowledge of HEPs Unavailability of HEW at Health post |
| P77 | Yilma, Y., et al. | 2020 | Mixed | Mixed | Oromia, Amhara, Southern Nations, Nationalities, and Peoples (SNNP), Tigray, Addis Ababa, Dire Dawa, Harar | Published Article | Reviews the overall implementation status, challenges  across the QITs, and the experience of selected QITs | Quality improvement | Multiple case study design to  learn about QI interventions | Quality Improvement Team | Poor coordination and leadership commitment. Loose linkage between HC staff and UHEP- |
| P78 | Estifanos, A. S., et al. | 2022 | mixed | Facility based | Amhara and Oromia regions | Published Article | Explore the reasons why healthcare providers intentionally falsify MNH data | Intentional data falsification by frontline MNCH workers | cross-sectional Qualitative study | health centre/department manager, the MCH focal person, the health information technicians (HITs), MNH clinical care providers (midwives, nurses and health officer) and HEWs. | Culture of silence masks widespread data falsification in healthcare, particularly inflated service numbers, underreported maternal deaths, and misclassified neonatal deaths. |
| P79 | Feysia, B., et al. | 2012 | NA | NA(It talks about various dimensions of the health workforce in Ethiopia) | National , Ethiopia | Grey | Bringing together the evidence to underpin policy discussions on HRH and help ﬁnalize the new HRH strategy | Health workforce challenges | A document reviews the current HRH situation in Ethiopia | studies on HRH and data from the FMOH and DHS | Limited set of skill among HEWs in essential maternal and child health services, including antenatal and postpartum care, injections, basic wound management, operating refrigerator. Supportive supervision is inadequate, characterized by infrequent visits, particularly in remote areas, and a focus on criticism rather than problem-solving. Limited resources for transportation |
| P80 | Vallières, F., et al. | 2020 | Mixed | Mixed | (Bangladesh,Malawi, Mozambique, Kenya), regional (Ethiopia) | Published Article | Identify factors that are associated with the motivation of CTC providers in the LMICs participating in the programme | motivation of community health workers | Qualitative study wth focus group discussions and interviews | HEWs | Organisational commitment, community commitment and work conscientiousness affects HEWs motivation |
| P81 | Nsibande, D., et al. | 2018 | Mixed | Facility based | Amhara, Benshangul, Oromia, SNNPR and Tigray | Published Article | Assess training, supervision and clinical mentoring of HEW regarding ICCM delivery | HEW training and supervision | A qualitative rapid appraisal study using focus group discussions and in-depth interviews | Federal Ministry of Health, Partners and researchers, nursing staff, UNICEF Country office, UNICEF Regional office, HEWs, Health Development Army | Low knowledge about HEP among community members, limited access to health posts, insufficient home visits by (HEWs, and urban-rural disparities |
| P82 | Posso, A., et al. | 2021 | Rural | Community based | Tigray, Amhara, Oromia, and the SNNP | Published Article | Investigate if exposure to a community-level health program delivered by Health Extension Workers (HEWs) lowers child labor | child labor | a children-focused panel survey | children aged between 5 and 15 | Inadequate resources, insufficient supportive supervision, poor quality of in-service training, lack of a standardized career development path for HEWs, low motivation, and high attrition rates. |
| P83 | Ye-Ebiyo, Y., et al. | 2007 | Mixed | Facility Based | Amhara Beni/G Harari Oromia SNNPR Tigra | Published Article | Make a clear needsassessment; assess access to CE, clearly map out and articulate priorities in and identify resources to undertake CE | HEWs access to information, continuing education and reference materials | A review of literature and documents and an in-depth field study | Document review, HP, HEWs and in-depth field study | HEWs limited access to information |
| P84 | Astale, T., et al. | 2023 | NA | NA(Review) | Low-and middle-income countries | Published Article | CHWs’ perceived workload in low-and middle-income countries (LMICs | Challenges of community health workers | A mixed-methods Systematic review: searched three electronic databases (PubMed, Scopus, and Embase) | Research articles | Lack of transport and High workload due to participation in multiple tasks |
| P85 | Schleiff, M. J., et al. | 2021 | NA | Review that discusses CHW | NA | Published Article | Explore a set of key considerations for training of CHWs in response to their enhanced and changing roles and provide actionable recommendations based on current evidence and case examples for health systems leaders and other stakeholders to utilize. | Training of CHWs | A focused review of relevant literature. | book chapter, peer-reviewed literature, WHO guidelines, and compendium | Gap in knowledge and skill among HEWs |
| P86 | Bilal, N. K., et al. | 2011 | NA |  | NA | Grey | Analyzing the role of HEWs in improving health access and covarage | access and coverage for the rural poor | Seems book chapter | Not stated | Inadequate training and skill levels of HEWs, cultural barriers, and low community engagement |
| P87 | Banteyerga, H. J. M. r. | 2011 | NA | NA(literature review) | Not stated | Published Article | sharing experience of HEP in Ethiopia | Role of HEP | Not specified in the document | Not stated | Model family graduation rates fell short of expectations due to prolonged training timelines caused by travel constraints and competing agricultural demands. Sustaining voluntary community health workers without financial incentives is challenging. HEW workload and burnout with development partners work. Lack clear career advancement |
| P88 | FMoH | 2016 | NA | Performance report for the health sector in Ethiopia | National , Ethiopia | Grey | Highlighting the three key features of quality and equity, universal health coverage (UHC), and transformation under four pillars of excellence of HSTP: (i) Health Service Delivery; (ii) Quality Improvement and Assurance; (iii)  Leadership and Governance; and (iv) Health System Capacity | Annual Performance Report | Health Sector Transformation Plan I: Annual Performance Report | Not stated | Inconsistent HDA implementation across regions due to managerial capacity limitation and poor collaboration among sector offices. |
| P89 | FMoH | 2015 | NA | NA(Strategic plan) | National , Ethiopia | Grey | Review of HEP performance |  | Health Sector Transformation Plan 2015/16 | Not stated | High turnover rate of HEWs |
| P90 | FMoH | 2018 | NA | NA | National , Ethiopia | Grey | Review of HEP performance | Special Bulletin | Special Bulletin: 20th Annual Review Meeting | Not stated | Medical supply stockout |
| P91 | FMoH | 2005 | NA | NA(Strategic plan) | National , Ethiopia | Grey | Review of HEP performance |  | Health Sector Strategic Plan (HSDP-III) | Not stated | inadequate infrastructure, limited access to essential drugs and supplies, a shortage of HEWS, high turnover rates, and insufficient community engagement |
| P92 | Mengesha, W., et al. | 2018 | Rural | Facility based | rural Southern Ethiopia | Published Article | Asees the impact of Mhealth on quality of data collection and reporting | timeliness and quality of health data | A mixed methods approach: Iterative process of intervention development, quantitative analysis of new registrations, and qualitative research with HEWs and their supervisors. | registrations, and HEWs and their supervisors | Technical limitation and increased workload related HMIS. |
| P93 | Zerfu, T. A., et al. | 2023 | Rural | community based | SNNPR, Gedeo zone | Published Article | Assess chalanges and experience of the Ethiopian rural health extension program | Challenges and experience of the Ethiopian rural health extension program | a longitudinal qualitative exploration linked to a larger cluster-randomized trial (RCT): in-depth interviews, focus group discussion, and passive observation | HEWs, district Health office representative, kebele administrators, pregnant and lactating mothers, family planning users, community leaders, husbands of pregnant or lactating mothers and elderly | Design flaws related to intervention packages, delivery methods, target populations and Female HEW struggle to engage the entire community |
| P94 | Kok, M. C., et al. | 2015 | NA | NA(literature review) | Low-and middle-income countries | Published Article | Determine factors determining the performance of community health workers | Performance of community health workers | Evidence from the literature | Research articles | CHW success hinges on community, economy, environment, and health system factors, including socio-cultural norms, safety, education, policies, resources, poor coordination, women's preference for home births HEWs demotivation. Geographical challenges, such as distance hinder CHW performance. Lack of resources and support |
| P95 | Kok, M. C., Kea, A. Z., Datiko, D. G., Broerse, J. E., Dieleman, M., Taegtmeyer, M., & Tulloch, O. | 2015 | Rural | Mixed | Sidama zone | Published Article | To assess HEW relationship with the community and the health sector in Ethiopia | Relationships of HEWs with the community and health sector | A qualitative study : FGD, semi-structured interview | HEWs, TBAs, health professionals and community members | Lack of support from kebele (local administrative unit) leaders,  communication barriers between HEWs and both the community and health sector Inconsistent support from local leaders and the Health Development Army (HDA)  Mismatch in expectations regarding the roles of HEWs and TBAs Non supportive supervision and lack of recognition  Inadequate training opportunities for HEWs |
| P96 | Medhanyie, A., Spigt, M., Dinant, G., & Blanco, R | 2012 | Rural | Facility based | Tigray region | Published Article | To evaluate the knowledge and performance of HEWs in providing antenatal and delivery care in Ethiopia | antenatal and delivery care | a cross-sectional study | HEWs | lack of basic infrastructures in health posts such as water supply, electricity, and waiting rooms for women in labour |
| P97 | Kok MC, et al | 2017 | NA(LMICs) | NA(review) | low- and middle-income countries | Published Article |  | performance of community health workers | A systematic review | Research articles | Model family training is taking longer than expected due to travel time between households and competing demands on family members' time for farming activities. Lacking curative services. The uptake of services provided by HEWs is limited by cultural beliefs, practices, and low literacy. Challenging geographical access to health posts and lack of infrastructure and equipment |
| P98 | Ogutu, M., Muraya, K., Mockler, D. et al | 2021 | NA(LMICs) | NA(review) | low- and middle-income countries | Published Article |  | performance of community health volunteer | A qualitative meta-synthesis review | Research articles | Lack of adequate training and Supportive supervision |
| P99 | Temesgen Ayehu, Gizachew Tadele Tiruneh, Chala Tesfaye, at al | 2025 | agrarian (rural) and pastoral settings | Facility-based, focused on Comprehensive Health Posts (CHPs) | Amhara, Oromia (Borena), Somali, and Afar | Published Article | To assess the readiness of comprehensive health posts (CHPs) to deliver obstetric care and  To explore the experiences of women and healthcare providers in delivering and receiving care in Ethiopia | HEP component: Reproductive, Maternal, Newborn, and Child Health (RMNCH) services, specifically maternal and newborn care (MCH) and childbirth services | Mixed, Qualitative and Quantitative | Postpartum women, Health Care providers including HEWs | Lack of electricity, frequent power interruptions, irregular water supply  Shortages of essential medicines, medical supplies, laboratory services, and delivery equipment  Staffing shortages, limited training, inadequate incentives or duty payments  Lack of capacity-building, career development opportunities, and attractive salaries for providers |
| P100 | Dawit Wolde Daka, Muluemebet Abera Wordofa, Mirkuzie Woldie | 2025 | Rural | Facility based | Amhara, Oromia, Tigray, SNNP | Published Article | To assess the readiness of health posts to deliver quality curative care for children under five in four regions of Ethiopia | Curative care for under-five children | Cross-sectional | 169 health posts  276 healthcare providers | Limited infrastructure  Stockouts of essential medicines (2–5 months average)  Weak infection prevention  Regional disparities in readiness  Inadequate supportive supervision and mentorship  Few HEWs received updated training in the past year |
| P101 | Mekides Geta, Geta Asrade Alemayehu, Wubshet Debebe Negash, | 2024 | Mixed | Facility based | Amhara | Published Article | To evaluate the implementation status of the Integrated Community Case Management (ICCM) program for common childhood illnesses in Gondar City, focusing on availability, compliance, and acceptability dimensions. | ICCM within the Health Extension Program – targeting childhood illnesses | Single-case study, concurrent mixed-methods evaluation | Under-five children and their caregivers in Gondar City  Health Extension Workers (HEWs), health facility providers, health managers, MCH coordinators, program stakeholders | Stock-outs of essential drugs and supplies  Infrastructure gaps  Shortage of HEWs and lack of refresher training  Weak supportive supervision and irregular performance review meetings  Poor compliance in assessment, classification, and treatment due to skill gaps  Caregivers dissatisfied with waiting area, cleanliness of health posts, and perceived competence of HEWs  Access barriers: long travel times and waiting times reduced acceptability |
| P102 | Tesfahun Hailemariam, Asmamaw Atnafu, Lemma Gezie, at al | 2024 | Rural | Community based | Amhara | Published Article | To assess health extension workers’ (HEWs) intentions to use the electronic community health information system (eCHIS) for health data management and service provision | eCHIS as part of the Health Extension Program (HEP) – focuses on health data capturing and use, MCH (maternal, newborn, and child health), reproductive health, referral linkage, and service delivery | Cross-sectional | HEWs | Lack of adequate resources for eCHIS implementation  Poor network access and electricity supply  Low financial investment  Variations in HEWs’ digital skills and knowledge  Need for continuous mentoring, supervision, and refresher trainings  Cross-sectional design limits cause-effect inference; limited to one zone (generalizability issue) |
| P103 | Daniel Nega, Samuel Ejeta Chibsa, Yared Nigusu, | 2024 | Rural | Facility based | Oromia | Published Article | To assess the level of job satisfaction and associated factors among rural health extension workers in Buno Bedele Zone | Job satisfaction among HEWs | Cross-sectional | HEWs | More than half of HEWs dissatisfied (only 45.5% satisfied)  Lack of residence/home near health posts  Limited transfer opportunities between kebeles  Inadequate medical supplies and equipment  Poor working environment  Limited or irregular technical support from supervisors  Organizational gaps: lack of recognition/incentives (64.2% did not receive recognition)  Risk of recall and social desirability bias due to self-reporting; absence of qualitative data for deeper exploration |
| P104 | Amlaku Nigusie Yirsaw, Gebeyehu Lakew, Eyob Getachew, | 2025 | NA (review) | NA (review) | National, all regions | Published Article | To estimate the pooled prevalence of job satisfaction and identify associated factors among Health Extension Workers (HEWs) in Ethiopia using systematic review and meta-analysis | Job satisfaction within the Health Extension Program (HEP), relevant to service delivery in MCH, FP, preventive and promotive services | Systematic review and meta-analysis | Research articles | Low pooled job satisfaction (46%; range 17–72%)  Lack of supportive supervision  Limited management support  Insufficient training opportunities  Resource shortages, workload imbalances, and salary disparities also identified as contextual challenges  Publication bias and heterogeneity among studies (limiting generalizability) |
| P105 | Tesfahun Hailemariam, Asmamaw Atnafu, Lemma Derseh Gezie, et al | 2025 | Rural | Community based | Amhara | Published Article | To determine the effect of an electronic community health information system (eCHIS) intervention on the maternal continuum of care in northwest Ethiopia. | Maternal continuum of care (completion of antenatal care, skilled birth attendance, and postnatal care within 48 hours) | Pre-post quasi-experimental study | Women who gave birth within the last 12 months preceding the survey and were permanent residents of the intervention district | Poor data quality and use in paper-based systems; low healthcare utilization; high maternal mortality |
| P106 | Hiwot Tesfa, Fentie Ambaw Getahun, Yihun Mulugeta Alemu | 2024 | Mixed, Urban, Rural | Community based | Amhara | Published Article | To assess the level of motivation and associated factors of the health development army (HDA) in the implementation of health extension packages | Motivation of Health Development Army (HDA) members.  All HEP components (e.g., MCH, EPI, sanitation, health education). | Cross-sectional | Health Development Army (HDA) | Low motivation overall (47.8% were motivated). Factors associated with lower motivation included: rural residence, age over 30, less than 4 years of work experience, low intrinsic job satisfaction, low community support, lack of supportive supervision, and not being recognized for their work. |
| P107 | Farhan Houssein Ali, Mansour Njah, David-Martin Millot, Kenza Hassouni | 2025 | NA (Multiple countries) | NA (review) | NA (Multiple countries) | Published Article | To examine the successes and challenges of Community Participation (CP) in Primary Health Care (PHC) and Health Programs (HP) in the IGAD region, focusing on the factors influencing and levels of community engagement. | MCH, Disease Prevention & Control, Health Systems Strengthening | Scoping Review | Peer-reviewed articles and grey literature | Unclear messaging, poor choice of channels, lack of feedback.  Lack of financial support, insufficient medical equipment, inadequate infrastructure, limited training materials.  Cultural beliefs, taboos, mistrust in health workers/institutions, language barriers, social exclusion and marginalization.  Political instability, armed conflicts, civil war  Long distances to health facilities, transportation issues, especially in rural/remote areas  Limited skills of CHWs, volunteer/staff fatigue, workload overload.  Weak engagement of state institutions, absence of policy standards and legislative frameworks, opaque selection processes for community representatives. |
| P108 | Amlaku Nigusie Yirsaw, Gebeyehu Lakew, Eyob Getachew, | 2025 | NA (review) | NA (review) | National, all regions | Published Article | To estimate the pooled prevalence of job satisfaction and identify associated factors among Health Extension Workers (HEWs) in Ethiopia using systematic review and meta-analysis | Job satisfaction within the Health Extension Program (HEP), relevant to service delivery in MCH, FP, preventive and promotive services | Systematic review and meta-analysis | Research articles | Low pooled job satisfaction (46%; range 17–72%)  Lack of supportive supervision  Limited management support  Insufficient training opportunities  Resource shortages, workload imbalances, and salary disparities also identified as contextual challenges  Publication bias and heterogeneity among studies (limiting generalizability) |
| P109 | Tesfahun Hailemariam, Asmamaw Atnafu, Lemma Derseh Gezie, et al | 2025 | Rural | Community based | Amhara | Published Article | To determine the effect of an electronic community health information system (eCHIS) intervention on the maternal continuum of care in northwest Ethiopia. | Maternal continuum of care (completion of antenatal care, skilled birth attendance, and postnatal care within 48 hours) | Pre-post quasi-experimental study | Women who gave birth within the last 12 months preceding the survey and were permanent residents of the intervention district | Poor data quality and use in paper-based systems; low healthcare utilization; high maternal mortality |
